# Clinical Findings in Healthy Dogs Fed With Diets Characterized by Different Carbohydrates Sources

**DOI:** 10.3389/fvets.2021.667318

**Published:** 2021-04-22

**Authors:** Manuela Gizzarelli, Serena Calabrò, Alessandro Vastolo, Giuseppe Molinaro, Ines Balestrino, Monica Isabella Cutrignelli

**Affiliations:** Department of Veterinary Medicine and Animal Production, University of Naples Federico II, Naples, Italy

**Keywords:** blood count, biochemical profile, taurine, pea, spelt, oats, pumpkin, amino acids

## Abstract

In recent years, pet owners have become more interested in the ingredients, and quality of pet-food, and several studies have demonstrated that feed management could affect healthy status. Recently, some authors indicated that commercial diets formulated without cereals, or using unconventional protein, and starch sources, can cause a reduction in taurine levels in both whole blood, and plasma. Nevertheless, the specific mechanism by means of which nutritional factors determine this reduction is not completely clear. Thirty neutered half-breed dogs were recruited at a kennel in the province of Naples (Italy) to investigate the influence of carbohydrates sources, and dietary density of nutrients on healthy status of dogs in terms of blood count, and biochemical parameters. The dogs were housed in the kennel and divided into three distinct groups. Three iso-energy, and iso-nitrogen commercial kibble diets (named GF1, GF2, and CB) with different protein, and carbohydrates contents, and carbohydrates sources were chosen for the trial. The chemical composition and amino acid profile of each of the three tested diets were analyzed. Moreover, blood samples of each dog were collected to evaluate the hematological and biochemical profiles. The taurine level was determined both on plasma and whole blood. The effect of the diets was analyzed statistically, and all tested diets were compared to the control one. There were significant differences between the three tested diets as regards their chemical composition. The concentrations of all amino acids seem to reflect protein content diets. The hematological profile resulted within the ranges considered physiological for the canine species for all subjects. Compared to the control diet, the three tested diets showed significant differences in blood count for MCHC and platelets. The biochemical profile showed significant differences between the diets, particularly their AST, fructosamine, lipase, and triglycerides values. The diets did not affect the blood and plasma taurine levels. They resulted in higher than optimal reserve levels. Preliminary results showed that the sources of carbohydrates and use of balanced diets affected only some biochemical parameters and did not alter the levels of taurine in healthy adult dogs.

## Introduction

In all developed countries, the companion animal population has gradually increased over the last 20 years, particularly in great urban centers (1). In Italy, about 39% of the population live with a cat and/or a dog (2). As a result, pet owners have been giving more attention to animal welfare and have become increasingly interested in the characteristics and production processes of commercial diets (3). The pet industry has produced several diets with particular ingredients or nutrients, suggested by specific claims, which could indicate beneficial effects, for example, grain-free diets which were formulated using tubers, and legumes as starch sources. Each carbohydrate source has a unique nutritional composition that could affect both the production process and use of nutrients (4). For instance, legume grains (pea and lentil) are rich in soluble dietary fiber (SDF, mean value: 26.9% of total dietary fiber, TDF), and proteins (CP, mean value: 23.87% DM) compared to cereals grains (e.g., corn and rice) (CP: 10.72% DM, SDF: 10.00% TDF) (5–7). Moreover, the amino acid profile of different protein sources varied significantly, legume protein is less rich in essential amino acids (e.g., taurine, L-carnitine) compared to protein sources of animal origin (6, 8, 9).

In 2018, the Food and Drug Administration (10) published a report on the possible link between grain-free diets and dilated cardiomyopathy related to taurine deficiency. Cardiomyopathy is characterized by a dilation of the left ventricle or both ventricles, in association with impairment of ventricular contractions (11) and it causes cardiac dysfunction (12). Moreover, some studies have indicated a correlation between taurine reduction due to the administration of grain-free diets and dilated cardiomyopathy in dogs (13–15).

Nevertheless, the results are controversial in the literature. A recent study conducted (16) on 86 Golden Retrievers observed significantly higher taurine values in whole blood in dogs fed a diet containing cereal compared to dogs fed grain-free diets. Significant differences in plasma taurine levels, however, were not observed.

On the contrary, Donadelli et al. (17) demonstrated significant increases in plasma taurine and whole blood taurine levels in Golden Retrievers fed grain-free diets for 26 weeks. Similarly, Pezzali et al. (18) found that grain-free diets had no effect on taurine levels. A recent review (19) highlighted some limits as far as current literature is concerned as regards identifying the specific nutritional causes of taurine deficiency and, consequently, dilatative myocardiopathy development in the dog.

The purpose of this study was to evaluate if the administration of three diets (two grain-free: GF1, and GF2 vs. one cereal-based: CB), over a medium-term period (5 weeks), formulated with different carbohydrate sources and amounts could affect blood profile, and biochemical parameters with particular regard to taurine levels in healthy dogs. We hypothesized that different carbohydrates sources could influence the healthy status of adult dogs.

## Materials and Methods

### Animals and Diets

The nutritional double-blind trial was performed at a private kennel located in the province of Naples (Italy). At the beginning of the trial, a veterinarian clinically examined 50 adult dogs and performed, hematological, biochemical, and parasitological tests (20) to exclude subjects with signs of pathologies. Subsequently, 30 adults, neutered, half-breed dogs (age 4 ± 1.20 years, weight 20.79 ± 6.38 kg, BCS 3.96 ± 0.95 on five point scale) were recruited. Each dog was housed in an individual box of 8 m^2^ (2 × 4) consisting of a closed rest portion (2 × 2), and an open common walking area for five adjacent boxes. Before the beginning of the study, and during the first days of each adaptation period, all dogs were submitted to copromicroscopic analysis for intestinal nematodes (*Trichuris, Toxocara, Toxascaris*, and *Ancylostomidae*), cardiopulmonary nematodes (*Angiostrongylus* and *Capillaria*), Cestode (*Dipylidium* and other *Taeniidae*), and Protozoa (*Giardia* and *Cystoisospora*). If they were found to be positive, they were immediately treated with specific deworming drugs.

After enrollment, the dogs were divided into three distinct groups (blue, red, and black), homogeneous for sex, age, weight, and BCS. For the experimentation, three commercial dry diets (Farmina-pet food, Nola, Italy) named GF1, GF2, and CB, respectively, were chosen and administered alternately to experimental groups following a Latin square scheme (3 diets × 3 groups); each experimental period had a total duration of 50 days (15 days of adaptation and 35 days of trial). Each diet was administered in a ratio of 110 kcal/kg^0.75^ of metabolizable energy (EM) (6).

The diets were characterized by similar energy densities (3,995 ± 4.73 kcal/kg), formulated mainly with the same protein source (chicken), but consisting of different carbohydrates sources (cereal grain vs. legume or tubers).

The ingredients of each diet were the following:

- Diet CTR: rice, beet pulp, poultry, and turkey meals, fat and oil, and minerals;
- Diet GF1: boneless chicken, dehydrated chicken protein, sweet potato, chicken fat, dried eggs, herring, dehydrated herring protein, fish oil (from herring), pea fiber, and dried carrot;
- Diet GF2: boneless chicken, dehydrated chicken protein, pea starch, chicken fat, dried pumpkin, dried eggs, herring, dehydrated herring protein, fish oil (from herring), pea fiber, and dried carrot;
- Diet CB: boneless chicken, dehydrated chicken protein, spelt, oats, chicken fat, dried eggs, herring, dehydrated herring protein, dried beet pulp, fish oil (from herring), and dried carrot.

### Diets Chemical Composition and Amino Acid Profile

An aliquot of 500 g for each diet was analyzed by means of near-infrared spectroscopy (NIRS DS 2005F, FOSS, Hilleroed, Denmark) to determine its chemical composition (21, 22). Total dietary fiber, and proportion soluble, and insoluble fractions were determined according to (23, 24). The diets were analyzed also to determine its amino acid profile using high-performance liquid chromatography (HPLC, Agilent Technologies 1290, California, CA, United States) according to Spitze et al. (25).

### Clinical Examination, Weight Checks, and Sampling

**Figure 1:**
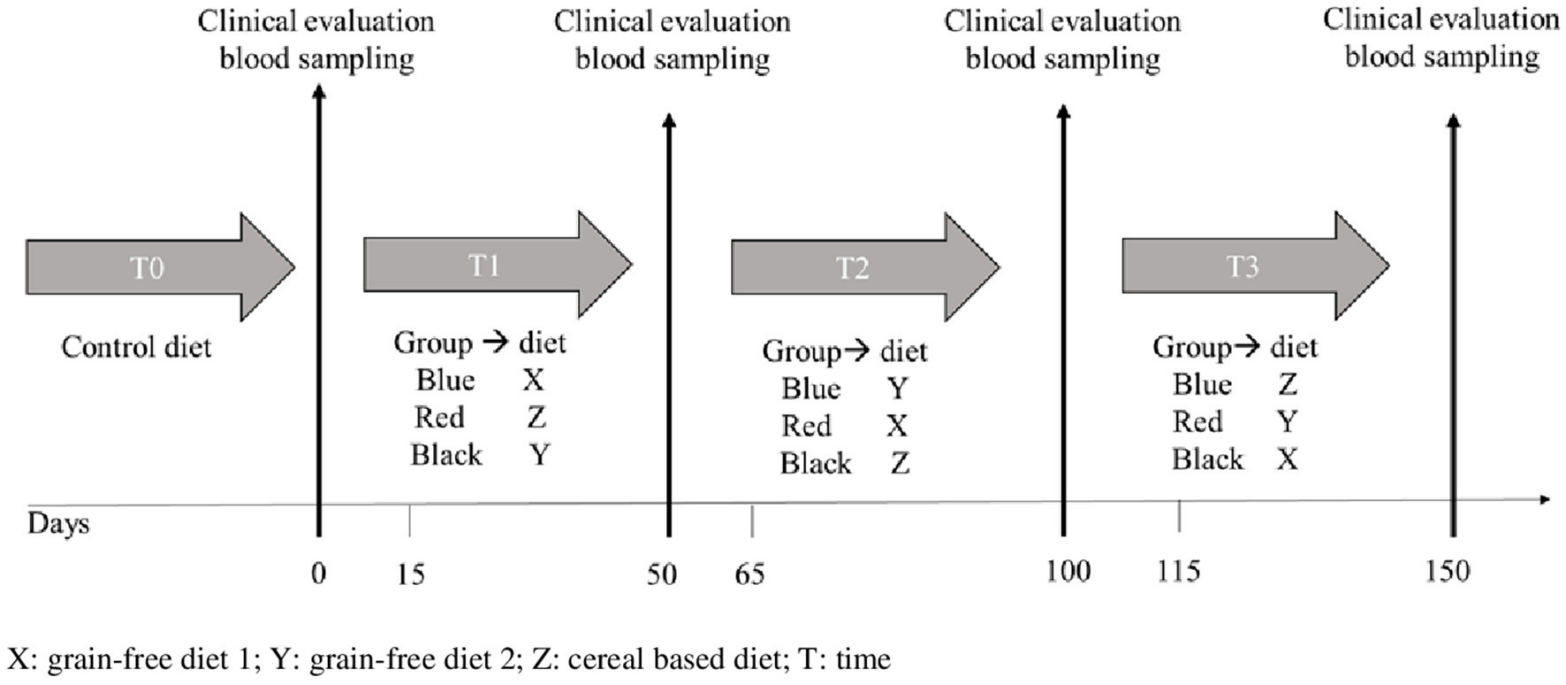
Study timeline.

At the beginning of each experimental period, the dogs were subjected to physical examination, weighed, and their body condition score (BCS) was evaluated. At recruitment and the end of each nutritional phases (Figure 1), fasted dogs were clinically evaluated and ~10 mL of blood was collected in three tubes:

- two with EDTA, one for determination of the blood count, and one for the dosage of plasmatic taurine;
- one for whole blood with separator gel from which to obtain the serum for determination of the biochemical profile.

The whole blood samples intended for the evaluation of the blood count were refrigerated, and quickly transported to the clinical analysis laboratory of the Department of Veterinary Medicine and Animal Production of the Federico II University of Naples. Each blood sample was analyzed using an impedance device to carry out an instrumental count (HeCo 5 Vet C, Real Time Diagnostic Systems; San Giovanni a Valdarno, Italy) after slow and constant mixing for 20 min.

At the kennel, to obtain the serum, the gel separator tubes were left at room temperature for about 15 min until the clot formed, then the samples were centrifuged for 10 min at a speed of 1,500 × g. The serum was stored at −80°C and subsequently sent on dry ice to a reference laboratory (Kornwestheim, Germany) where the following parameters were determined using a Beckman biochemical analyzer (Beckman Coulter AU5400; Olympus America, Melville, NY, USA): Globulin; Total protein (TP); Albumin (Alb); Alkaline phosphatase (AP); Glutamic Pyruvic Transaminase (GPT); Alanine Transaminase (ALT); Gamma-glutamyl transferase (GGT); Aspartate Transferase (AST); Glutamate dehydrogenase (GLDH); Fructosamine (Fr); Glucose (GLU); α-amylase; Lipase (LP); Sodium (Na); Potassium (K); Calcium (Ca); Chloride (Cl); Phosphorus (P); Magnesium (Mg); Cholesterol (Col); Triglycerides (Tri); Creatinine (Crea); BUN, Creatine kinase (CK).

At the end of each phase from five dogs per group blood were sampled to determine taurine on whole blood; after sampling blood was collected in a tube containing lithium heparin. Subsequently, it was gently mixed and stored at a temperature of −80°C up to the shipment on dry ice to the Amino Acid Laboratory (University of California, Davis, CA, USA). Whole blood taurine concentrations were determined using a Beckman 6,300 amino acid analyzer (Beckman Industries, Palo Alto, CA, USA).

Taurine in plasma was determined by reversed phase chromatography hyphenated to a triple quadrupole mass spectrometer (LC-MS/MS Sciex API4000QTRAP plus Agilent HPLC and CTC PAL autosampler, Santa Clara, CA, USA) (26).

### Statistical Analysis

The effects of the diets were analyzed by one-way analysis of variance (ANOVA). Tukey's HSD test was used when significant differences were observed.

To compare each diet with the control one, the Dunnett test was used, which allows multiple comparisons. All statistical analyses were performed using the software JMP 14 (SAS Institute, NC, USA).

## Results

Table 1 shows the chemical composition of the diets used during the trial. All statistical analyses were referred comparing the diets. GF1 showed the highest value in protein, and ash (*P* < 0.01 and *P* < 0.05, respectively). The energy nutrients (protein, carbohydrates, and lipids) of GF1, GF2, and CB diets were equally proportioned. Moreover, the three diets tested resulted in higher protein and energy levels than the CTR one, which showed higher carbohydrates content. Total dietary fiber (TDF) content of GF1 was significantly (*P* < 0.05) lower than CB one (data not reported 76.78 vs. 83.73 vs. 94.30 g/kg as is, for GF1, GF2, and CB, respectively). Moreover, all diets showed higher incidence of soluble fiber than insoluble fiber (60.65; 58.61; and 63.15% TDF, respectively).

**Table 1:** Diets chemical composition (g/kg as is) and energy content (ME kcal/kg as is).

**Nutritional characteristics**	CTR^*^	**GF1**	**GF2**	**CB**	**RMSE**
Crude Protein	240	363^A^	316^B^	317^B^	19.1
Total fat	100	185	193	194	51.5
Crude Fiber	38.0	23.0	23.7	24.7	9.41
Ash	99.0	65.0^a^	60.0^b^	60.3^b^	20.3
Metabolizable Energy^*^	3,200	3,999	3,990	3,997	140

^*^Chemical composition of control diet as reported in the label; GF1, grain-free diet 1; GF2, grain-free diet 2; CB, cereal based diet; along the row the capital letters indicate differences for P < 0.01 and P < 0.001; lowercase indicated differences for P < 0.05. RMSE, root mean square error. The statistical comparison was performed between GF1, GF2, and CB diets. ^*^ME, calculated according to the predictive equation indicated by NRC 2006 (6).

Table 2 shows the amino acid concentrations of the three diets. In all cases, the most present amino acids were glutamic acid, glycine, alanine, arginine, aspartic acid, and lysine. The concentrations of all amino acids seem to reflect protein content of the diets.

**Table 2:** Amino acids profile of diets used during the trial.

	**GF1**	**GF2**	**CB**	**GF1**	**GF2**	**CB**	FEDIAF^*^
	**% as is**			**g/1,000 kcal ME**			
Protein	36.3	31.6	31.7	97.19	85.25	84.91	45.00
Alanine	2.19	1.99	1.90	5.86	5.37	5.09	NA
Arginine	2.11	1.88	1.80	5.65	5.07	4.82	1.30
Aspartic acids	2.83	2.80	2.26	7.58	7.55	6.05	NA
Glutamic acids	4.29	4.17	4.54	11.5	11.3	12.2	NA
Glycine	3.24	2.67	2.86	8.67	7.20	7.66	NA
Histidine	0.76	0.72	0.68	2.03	1.94	1.82	0.58
Hydroxyproline	1.01	0.71	0.95	2.70	1.92	2.54	NA
Isoleucine	1.13	1.12	0.97	3.03	3.02	2.60	1.15
Leucine	2.32	2.20	2.07	6.21	5.94	5.54	2.05
Lysine	2.08	2.11	1.65	5.57	5.69	4.42	0.25
Phenylalanine	1.30	1.22	1.18	3.48	3.29	3.16	1.35
Proline	2.20	1.77	2.21	5.89	4.78	5.92	NA
Serine	1.43	1.32	1.29	3.83	3.56	3.46	NA
Threonine	1.27	1.22	1.08	3.40	3.29	2.89	1.30
Tyrosine	0.87	0.84	0.77	2.33	2.27	2.06	NA
Valine	1.54	1.48	1.35	4.12	3.99	3.62	1.48
Cystine	0.42	0.37	0.43	1.12	1.00	1.15	NA
Methionine	1.10	1.00	0.93	2.95	2.70	2.49	1.00
Tryptophane	0.33	0.33	0.32	0.88	0.89	0.86	0.43
Taurine	0.22	0.23	0.18	0.59	0.62	0.48	NA

* *Nutritional values and FEDIAF (2020) (27) recommendation based on a metabolizable energy requirement of 110 kcal ME/kg0.75; GF1, grain-free diet 1; GF2, grain-free diet 2; CB, cereal based diet; NA, not applicable*.

The results of the blood count are reported in Table 3. All recorded values fall into the relative physiological range for canine species. The analysis of variance did not show differences between the diets. Nevertheless, the Dunnett test indicated significant (*P* < 0.01) differences between CTR diet and each tested one for MCHC, which resulted in 1 g/dL lower in CTR than GF1, GF2, and CB. Moreover, platelet values resulted significantly (*P* < 0.05) higher for CTR compared to that of CB diet (328 vs. 287 K/uL, respectively).

**Table 3:** Blood profile of dogs in function of administered diet.

		**Diets**					**CTR vs**.		
**Items**	**Units**	**CTR**	**GF1**	**GF2**	**CB**	**RMSE**	**GF1**	**GF2**	**CB**
RBC	M/uL	6.88	7.00	6.95	6.76	0.64	NS	NS	NS
WBC	K/uL	12.8	11.4	12.6	13.00	2.42	NS	NS	NS
Hgb	g/dL	16.4	15.8	16.3	16.2	1.77	NS	NS	NS
Hct	%	48.1	47.9	47.3	47.3	4.57	NS	NS	NS
MCV	fL	69.2	68.4	68.0	68.7	2.80	NS	NS	NS
MCH	Pg	23.8	24.0	23.9	24.1	1.16	NS	NS	NS
MCHC	g/dL	34.4	35.1	35.1	35.1	0.70	^**^	^**^	^**^
Plt	K/uL	328	298	306	287	42.0	NS	NS	^*^

CTR, control; GF1, grain-free diet 1; GF2, grain-free diet 2; CB, cereal based diet; RBC, red blood cells; WBC, white blood cells; Hgb, hemoglobin; Hct, hematocrit; MCV, medium corpuscular volume; MCH, medium corpuscular hemoglobin; MCHC, mean corpuscular hemoglobin concentration; Plt, platelets; ^*^, ^**^, P < 0.05 and P < 0.01, respectively; NS, not significant; RMSE, root mean square error.

Table 4 shows biochemical values. The nutritional treatment affected only a few parameters. The analyses of variance evidenced that the dogs showed significantly higher (*P* < 0.01) values as regards AST, fructosamine, lipase, and triglycerides when fed CTR diets. The Dunnett test evidenced the differences between CTR diets vs. each tested diet more clearly, in particular for triglycerides which were about double when CTR diet was administered.

**Table 4:** Biochemical profile of dogs in function of diets.

		**Diets**					**CTR vs**.		
**Items**	**Units**	**CTR**	**GF1**	**GF2**	**CB**	**RMSE**	**GF1**	**GF2**	**CB**
Gl	g/L	37.6	35.8	34.7	36.0	5.47	NS	NS	NS
PT	g/L	66.9	67.1	64.9	65.2	6.25	NS	NS	NS
Alb	g/L	29.4	28.9	30.3	29.6	1.62	NS	NS	NS
AP	U/L	38.2	30.5	31.3	30.7	11.8	NS	NS	NS
Crea	μmol/L	80.7	72.6	74.2	76.0	12.1	NS	NS	NS
BUN	mmol/L	6.81	5.74	6.11	6.57	1.46	NS	NS	NS
CK	U/L	113	106	103	102	27.0	NS	NS	NS
ALT	U/L	39.3	39.1	38.5	39.3	8.44	NS	NS	NS
GGT	U/L	3.11	3.04	2.96	3.19	0.97	NS	NS	NS
AST	U/L	40.0^A^	33.9^AB^	30.5^B^	31.2^B^	7.61	^*^	^***^	^*^
GLDH	U/L	3.05	3.08	3.12	3.42	1.29	NS	NS	NS
Fr	μmol/L	204^A^	187^B^	186^B^	188^B^	9.93	^***^	^***^	^***^
Glu	mmol/L	5.03	4.79	4.94	4.95	0.64	NS	NS	NS
α-amylase	U/L	781	763	759	797	109	NS	NS	NS
LP	U/L	133^A^	67^B^	65^B^	69^B^	15.9	^***^	^***^	^***^
Chol	mmol/L	4.48	4.93	4.78	4.99	0.86	NS	NS	NS
Tri	mmol/L	0.88^A^	0.59^B^	0.58^B^	0.56^B^	0.17	^***^	^***^	^***^

CTR, control diet; GF1, grain-free diet 1; GF2, grain-free diet 2; CB, cereal based diet; Gl, Globulin; PT, Total protein; Alb, Albumin: AP, Alkaline phosphatase; Crea, Creatinine; CK, Creatine kinase; ALT, Alanine Transaminase; GGT, Gamma-glutamyl transferase; AST, Aspartate Transferase; GLDH, Glutamate dehydrogenase; Fr, Fructosamine; Glu, Glucose; LP, Lipase; chol, Cholesterol; Tri, Triglyceride along the row the capital letters indicate differences to P < 0.01 and P < 0.001; NS, not significant, ^*^, ^***^: p < 0.05, p < 0.001, respectively; RMSE, root mean square error.

Table 5 shows the mineral profile. All parameters are within the physiological ranges for healthy dogs. With both statistical analyses, significant differences were observed for potassium, phosphorus, and magnesium which were always significantly higher in CTR than in GF1, GF2, and CB diets.

**Table 5:** Mineral profile (mmol/L) of dogs in function of diets and comparison of each diet to control one.

	**Diets**					**CTR vs**.		
**Items**	**CTR**	**GF1**	**GF2**	**CB**	**RMSE**	**GF1**	**GF2**	**CB**
Na	147	147	146	146	2.40	NS	NS	NS
K	4.92^A^	4.55^B^	4.41^B^	4.59^B^	0.40	^*^	^***^	^*^
Ca	2.43	2.45	2.44	2.44	0.14	NS	NS	NS
Cl	111	111	110	111	2.71	NS	NS	NS
P	1.33^A^	1.37^A^	1.24^AB^	1.18^B^	0.18	NS	NS	^*^
Mg	0.90^A^	0.81^B^	0.80^B^	0.82^B^	0.08	^***^	^***^	^**^

CTR, control diet; GF1, grain-free diet 1; GF2, grain-free diet 2; CB, cereal based diet; Na, Sodium; K, Potassium; Ca, Calcium; Cl, Chloride; P, Phosphorus; Mg, Magnesium; along the rows the capital letters indicate differences to p < 0.01 and p < 0.001; NS, not significant, ^*^, ^**^, ^***^, p < 0.05, p < 0.01, p < 0.001, respectively; RMSE, root mean square error.

In Table 6, whole blood and plasma taurine levels are shown. In both cases, taurine levels were not significantly affected by the administered diet, even if the CTR diet showed the lowest value of both parameters.

**Table 6:** Blood (*n* = 60) and plasma (*n* = 120) taurine levels of dogs in function of diets.

		**Diets**					**CTR vs**.		
**Taurine**	**Units**	**CTR**	**GF1**	**GF2**	**CB**	**RMSE**	**GF1**	**GF2**	**CB**
Whole blood	μmol/l	288	316	316	318	51.3	NS	NS	NS
Plasma	μmol/l	101	128	127	125	32.8	NS	NS	NS

*CTR, control diet; GF1, grain-free diet 1; GF2, grain-free diet 2; CB, cereal based diet; NS, not significant; RMSE, root mean square error*.

## Discussion

All diets showed nutritional characteristics able to satisfy the nutritional requirement of adult dogs located in a kennel (6). In particular, the amount of crude protein, total fat, and metabolizable energy fall into the levels recommended by FEDIAF 2020 (27). During the experimental period, we did not find refusals probably due to the high palatability of all diets. Considering that no significant differences were observed as regards live weight, and body condition score (final live weight 21.59 ± 5.70 kg; BCS 3.97 ± 0.81 on a five-point scale) we can assert that the amount of feed administered was correctly calculated in all groups (28).

The amount of essential amino acids (g/1,000 kcal) in the three tested diets were about double the minimum levels recommended by FEDIAF (27) for an adult dog which has metabolized energy requirement equal to 110 kcal/kg^0.75^. All experimental diets are able to fully satisfy even the aforementioned nutritional requirements for a dog with a lower energy necessity (95 kcal/kg^0.75^), also considering an apparent availability of 70% (29).

There were no significant changes observed in the hematological profiles within the diets. All dogs showed the blood count values within the ranges considered physiological. The only exceptions were recorded for two factors, MCHC (CTR vs. GF1, GF2, and CB, *P* < 0.01) and platelets (CTR vs. CB, *P* < 0.05). Actually, from our study, whether these findings are coincidental or just a trend cannot be established, nor it is possible to give a definitive explanation. It is essential to underline that, even if there were significant differences, values for MCHC and platelets were both within ranges. Considering the absence of clinical signs or clinicopathological alterations and the negativity as regards the mainly canine vector-borne diseases, it seems likely that the results were due to any pathological cause. There is very little data available concerning the influence of diet on hematological parameters (30, 31) that could clarify the differences that resulted from our trial. However, it is interesting to note that in the study by Anturaniemi et al. (31), higher erythrocyte counts, and hemoglobin levels occurred in dogs fed with a high protein diet when as compared to those fed on the lowest protein diet. In the present study, the increased values of MCHC in dogs fed with diet which was richer in proteins than the control may corroborate the same trend, also considering that dietary protein may play a role in maintaining appropriate red blood cell indices (32). Clearly, further study is needed to better determine which dietary factor is responsible.

Some pre-analytical and analytical bias cannot be excluded, even if points sampling procedures and processing were standardized at all times (33, 34). Moreover, no specific trend for platelets in dogs or diets during the trial was observed. Furthermore, platelets numbers could vary in different physiological conditions, above all due to the presence of platelet clumps, often caused by sampling procedures and collection (35).

In addition, the biochemical profile also falls into the physiological range for canine species. The differences related to fructosamine, lipase, and triglycerides obtained by the Dunnett test seem to indicate a dietary effect on carbohydrates and lipid metabolism. In particular, the redaction of these parameters observed when the dogs were fed GF1, GF2, and CB diets could be related to the different proportions of carbohydrates, lipids and proteins in these diets compared to CTR. The sources of carbohydrates used in the formulation of the control diet showed a higher content of nitrogen free extractives than the others (NFE: 42.3 vs. 30.83; 34.83, and 34.60% as is, respectively, for the CTR, GF1, GF2, and CB diets), while the fat and protein contents are significantly lower in the control diet than those used for the trial. The reduction of AST observed with all diets than the control could be indicative of lower hepatic stress. Aspartate aminotransferase is an enzyme that is found mainly in the liver and heart and, in lower concentrations, in the kidneys and muscles and low levels of AST are indicative of good health, while when the liver or muscle cells are damaged, the enzyme is released into the blood in higher quantities. Although elevated serum levels of AST could be considered a sign of a hepatic injury or disease, concomitant with other variations of hematological parameters (e.g., lower ALT values) and other clinical signs (36). In our case, clinical signs of hepatic injury were not observed, and all parameters could be considered physiological. Nevertheless, higher metabolic activity in the liver could be indicated by the higher AST values registered with CTR, and GF1 diets (37).

Another important aspect may have also concerned the carbohydrate sources used. While the CTR diet was formulated with rice (source of starch) and beet pulp, which mainly provides insoluble fiber, in the other three diets carbohydrates sources such as pea starch, spelt and oats, which are characterized by low glycemic index, and carrot, squash and pea fiber as sources of dietary fiber were used (38, 39). These ingredients guarantee a greater intake of soluble dietary fiber, able to modulate the post-prandial glycemic response. Moreover, these different dietary components guarantee the maintenance of the balance of saprophytic bacterial populations of the large intestine as it is fermented here, thereby producing short-chain fatty acids (40), in particular butyrate, which is considered the main energy source for erythrocytes and colonocytes. Dietary fiber has been indicated as a nutritional factor able to modify lipid absorption reducing, directly and indirectly, bile acid reabsorption (41). The decreasing of triglycerides observed with diets GF1, GF2, and CB could be due to limited absorption of triglycerides in the small intestine (41, 42). The reduced lipid absorption was confirmed by the significant reduction of pancreatic lipase production. Indeed, Stock-Damge' et al. (43), administering a diet supplemented with 5 g/d of wheat bran for 4 weeks, observed significantly higher (*P* < 0.05) pancreatic secretion and lower (*P* < 0.05) lipase concentration.

The significantly higher serum concentration of potassium, phosphorus, and magnesium registered when the dogs fed control diet could be related to the higher concentration of phosphorus in this diet (12 vs. 8 mg/kg). On the other hand, the higher value of phosphorus level in serum of dog fed GF1 diet could be related to the higher bioavailability of this element that mainly derived from animal sources in this diet. Moreover, CTR and GF1 diets were characterized by the higher Ca:P ratio (1.50; 1.25; 1.12 and 1.12, CTR; GF1; GF2; CB diet, respectively) (6).

Although no statistically significant differences were observed, there was an increased value in both parameters compared to the initial values of 288.27 and 101 nmol/l, recorded with the control diet. Another interesting aspect is that in all cases the taurine levels were higher than the optimal reserve levels indicated by FEDIAF (27) (>40 nmol/l in plasma and >200 nmol/l in whole blood) and by University researchers California (44) (>70 nmol/ml in plasma and >250 nmol/ml in whole blood), regardless of the dilatative cardiomyopathy risk in adult dogs.

The literature concerning the effect of diet on taurine concentration in blood and serum is controversial. Delaney et al. (45) observed that the whole blood taurine concentration was lower in dogs fed whole grain rice, rice bran, or barley. Freid et al. (46) in a retrospective study on dogs affected by dilatative myopathies, observed that dogs fed a non-traditional diet (grain-free contained novel ingredients such as peas or lentils as the main component) showed status improvement after their diet was changed.

Donadelli et al. (17) did not observe a reduction in plasma amino acids and taurine status when Labrador Retrievers were fed with a commercial grain-free diet after 26 weeks.

In our study, conducted on healthy dogs, increased taurine levels in whole blood and plasma were observed after 5 weeks of the administration of three diets. The control diet (used before the trial) could be defined as a traditional diet (grain inclusive with rice and beet pulp). While the three diets tested showed particular nutritional characteristics, and carbohydrates ingredients: GF1 (grain-free with sweet potato, pea fiber, dried carrot); GF2 (grain-free with pea starch, dried pumpkin, pea fiber, dried carrot); and CB (grain inclusive with spelt, oats, dried beet pulp, dried carrot). It seems possible to affirm that the relative proportions of the nutrients in the diets rather than the use of novel ingredients could affect taurine level. Indeed, all tested diets are characterized by the use of high-quality protein sources (dehydrated and fresh chicken, herring, and eggs), and high levels of protein inclusion allow protein and amino acids requirements to be satisfied and, consequently, the taurine status.

Moreover, the relative lower root means square error of taurine in whole blood compared to plasma one confirms the previous observation (45) that taurine has greater stability in whole blood.

## Conclusion

Our preliminary results showed that only a few hematological parameters were affected when balanced diets were administered to healthy dogs. The sources of carbohydrates (starch and dietary fiber) and the appropriate equilibrium between energy nutrients (e.g., protein, fat, and starch) could modify the indicators of lipid, and carbohydrate metabolism (AST, fructosamine, lipase, triglycerides) and improve liver function.

## Data Availability Statement

The raw data supporting the conclusions of this article will be made available by the authors, without undue reservation.

## Ethics Statement

All the procedures used in the study have been approved by the Ethics Committee for the care and use of animals of the University of Naples Federico II in accordance with local and national regulations and guidelines (Legislative Decree 26 of 04/03/2014).

## Author Contributions

MC: conceptualization. AV, GM, and IB: formal analysis. IB and AV: methodology and data curation. SC and AV: statistical analysis. MG and GM: clinical visitation. MG and AV: writing—original draft. SC and MC: writing—review and editing. MC and MG: supervision. All authors contributed to the article and approved the submitted version.

## Conflict of Interest

The authors declare that the research was conducted in the absence of any commercial or financial relationships that could be construed as a potential conflict of interest.
